# Craniofacial Morphology and Cephalometric Analysis of Children with Tall Stature and Overgrowth Due to Molecular Genetic Etiology: A Scoping Review

**DOI:** 10.3390/genes17101167

**Published:** 2026-09-23

**Authors:** Nickolas Ziakas, Myrsini Chamakioti, Iphigenia Gintoni, Demetrios J. Halazonetis, George P. Chrousos, Christos Yapijakis

**Affiliations:** 1 Unit of Orofacial Genetics, 1st Department of Pediatrics, National Kapodistrian University of Athens, “Aghia Sophia” Children’s Hospital, 115 27 Athens, Greece; nickolas_ziakas@hotmail.com (N.Z.); m.chamakioti@gmail.com (M.C.); iph.gintoni@gmail.com (I.G.); 2 Choremion Laboratory, University Research Institute of Maternal and Child Health and Precision Medicine, “Aghia Sophia” Children’s Hospital, 115 27 Athens, Greece; chrousge@med.uoa.gr; 3 Department of Orthodontics, School of Dentistry, National and Kapodistrian University of Athens, 115 27 Athens, Greece; dhal@dhal.com

**Keywords:** tall stature, gigantism, genetics, genetic syndromes, craniofacial morphology, cephalometric radiograph, growth hormone/insulin-like growth factor 1 axis, epiphyseal growth plate, molecular pathways

## Abstract

Background: Tall stature is a multifactorial phenotypic trait encountered in the general pediatric population; however, it could constitute a manifestation of an underlying genetic and/or endocrine disorder. Mutations in genes involved in gene transcription, genomic imprinting and intracellular vesicle trafficking may lead to dysregulation of certain cellular pathways associated with different genetic syndromes with overgrowth and tall stature. Objective: Considering the facial changes that growth hormone surplus induces, it would be reasonable to hypothesize that the maxillofacial phenotype is also affected in certain genetic conditions causing excessive linear growth. The aim of this review is to assess the cephalometric data for certain genetic and/or endocrine diseases causing tall stature and overgrowth in children and discuss the potential role of cephalometric analysis in diagnosis of such phenotypes and better understanding of the underlying causing molecular mechanisms. Design: A literature search was conducted in the PubMed electronic database. The scientific articles that provided cephalometric data for genetic syndromes and conditions associated with tall stature or overgrowth were included. The following keywords were utilized: tall stature cephalometry, overgrowth syndromes cephalometry, gigantism cephalometry, hemihyperplasia cephalometric, hemihypertrophy cephalometric, Simpson–Golabi–Behmel cephalometric, Prader–Willi cephalometry, and Marfan and Klinefelter cephalometry. Results: Although the number of studies found remains limited (*n* = 37), the consensus findings indicate that the growth of the craniofacial complex of patients with genetic syndromes causing tall stature is affected. The cephalometric measurements assessing the cranial base, the jaws and the facial soft tissue differ from the norm and are suggestive of irregularities in both the sagittal and vertical planes. Conclusions: As the literature in this field is particularly rare, more data are required to establish relations between excessive linear growth and the maxillofacial phenotype exhibited in certain genetic and/or endocrine syndromes. Nevertheless, lateral cephalometric radiography is an extensively utilized imaging method that could potentially serve as a promising tool in the early diagnosis, phenotype–genotype comparative research and better understanding of the underlying causing molecular mechanisms of conditions associated with tall stature.

## 1. Introduction

Stature is a multifactorial phenotypic trait influenced by genetic ancestry and environmental factors. There is a significant variation in population-level stature across ethnic and racial groups with groups of northern European descent being on average the tallest and east Asian groups being the shortest. Average heights of male adults in the U.S.A. reportedly appeared to follow a gradient of White males (≈5 ft 9.8 in or ≈1.77 m), Black males (≈5 ft 9.1 in or ≈1.75 m), Asian (≈5 ft 6.8 in or ≈1.70 m) and Hispanic males (≈5 ft 6.7 in or ≈1.69 m) [1]. The above patterns have been confirmed by genome-wide association studies, showing that polygenic height variants differ in frequency across ancestries [2]. Furthermore, it has been observed that migration to better-resourced environments can substantially increase height in subsequent generations, underscoring the important role of the interplay between genetics and ambience [3]. Nutrition represents a pivotal modulating factor of childhood linear growth, since chronic undernutrition leads to stunting (height-for-age below −2 SD) via impaired growth plate function and delayed maturation, while abundant caloric and protein intake supports optimal stature gain. As demonstrated by longitudinal studies, early-life malnutrition causes height deficits largely irreversible after age 2 years [4]. On the other hand, overnutrition or obesity may accelerate growth velocity through insulin-like effects on insulin-like growth factor 1 (IGF1), but extreme cases rarely cause pathologic tall stature without an endocrine involvement [5].

The terms ‘tall stature’, ‘overgrowth’, and ‘gigantism’ are sometimes used interchangeably in clinical practice and published articles, although they denote different phenotypic and etiologic entities. The term ‘tall stature’, also known as excessive linear growth, is typically defined as a height more than two standard deviations above the mean (or above the 97th percentile) for age and gender on standardized growth charts from the World Health Organization (WHO) and Centers for Disease Control and Prevention (CDC) [6,7]. Although familial or constitutional tall stature accounts for the majority of cases, excessive linear growth may prompt medical evaluation for an underlying genetic, endocrine or another identifiable or even unidentifiable disorder [8].

On the other hand, the term ‘overgrowth’ broadly describes abnormal tall stature and is used to define the following three distinct phenotypical patterns: (a) prenatal overgrowth; large size at birth, often due to maternal diabetes or overgrowth syndromes such as Beckwith–Wiedemann, (b) postnatal overgrowth; rapid growth beginning in childhood or even adolescence, often due to endocrine causes, but also due to genetic conditions like Marfan syndrome, homocystinuria, or sex chromosome aneuploidies, and (c) segmental overgrowth; excessive growth limited to part of the body, usually due to mosaic mutations, most often in the PI3K/AKT/mTOR pathway [9]. Finally, ‘gigantism’ is abnormal tall stature caused by excessive growth hormone secretion before the epiphyseal growth plates fuse. In particular, acromegaly is excessive growth hormone secretion after epiphyseal closure, causing enlargement of the hands, feet, and facial features [10].

Linear growth is controlled by a variety of endocrine, paracrine, and extracellular matrix factors. These regulators coordinate chondrogenesis at the epiphyseal growth plate and thereby the longitudinal bone development. A disrupted function of these factors can be clinically obvious as deviations from normal stature, extending from proportionate or disproportionate short stature to marked tall stature [11].

Children with excessive tall stature may encompass a smaller proportion of referrals to pediatric endocrinology clinics compared to those with growth failure; however, prompt evaluation is critical because of the possible existence of an underlying endocrine, neoplastic, or genetic disease [11]. Among pathological causes, the prototypical driver of extreme linear overgrowth prior to epiphyseal fusion is the childhood-onset growth hormone (GH) excess due to pituitary hyperplasia or somatotroph adenomas—classically termed gigantism. Moreover, tall stature of genetic etiology also includes monogenic conditions such as *AIP*-related familial isolated pituitary adenomas, *GPR101*-related X-linked acrogigantism [12], and other syndromes such as McCune–Albright and multiple endocrine neoplasia. In contrast to this, constitutional or idiopathic tall stature is characterized by normal endocrine profiles and normal pituitary function and represents a diagnostic category that must be distinguished from GH-driven overgrowth.

During childhood the craniofacial skeleton is particularly susceptible to hormonal and genetic influences, as the cranial base synchondroses are still active [13]. For this reason, identification of such typical features through lateral cephalometric measurements can contribute to the detection of a broad range of growth abnormalities. Studies in children with short stature of genetic etiology indicate that disturbances of systemic growth are paralleled by consistent changes in craniofacial morphology [14]. In a similar manner, the characteristic craniofacial features of adults with acromegaly and children with GH excess, such as mandibular prognathism, enlarged facial dimensions and altered maxillomandibular proportions, suggest that genetically associated overgrowth states may be accompanied by distinct morphologic characteristics of the craniofacial complex [15].

However, it is unclear in the current literature if there is a specific pattern of the craniofacial data that accompany each tall stature condition. For that reason, a scoping review was conducted in order to systematically map the research done in this area, as well as to identify any existing gaps in knowledge. The formulated research questions included what is currently available in the literature about lateral cephalometric characteristics in children with tall stature of genetic and/or endocrine etiology, as well as what is the role of these craniofacial traits as markers for earlier recognition and improved genetic and/or endocrine evaluation of pediatric tall stature.

### 1.1. The Multifactorial Etiology of Tall Stature

The epiphyseal growth plate (EGP) is the key biological structure responsible for longitudinal bone growth through the process of chondrogenesis and ultimately determines adult stature. About twenty years ago, excessive linear growth was thought to result primarily from abnormalities of the growth hormone/insulin-like growth factor 1 (GH/IGF1) axis. However, advances in the field have overturned this concept and have revealed a multitude of biological mechanisms that regulate growth plate homeostasis. Chondrogenesis within the EGP is delicately orchestrated by the interaction of numerous intrinsic (endogenous to the growth plate) and extrinsic (exogenous to the growth plate) factors that act either as stimulators or inhibitors of chondrocyte metabolism [10]. These regulatory mechanisms include endocrine, paracrine, and nutritional factors, physical influences, extracellular fluid parameters, proinflammatory cytokines, extracellular matrix molecules, and intracellular signaling pathways [10]. Genetic or acquired disturbances affecting any of these biological processes may enhance EGP activity and result in excessive skeletal growth, manifesting clinically as tall stature [10]. Consequently, tall stature should not be regarded solely as a consequence of increased GH secretion but rather as the phenotypic manifestation of diverse biological mechanisms affecting growth plate function and therefore skeletal development [10].

It is well known that as a multifactorial phenotypic trait, stature is influenced by interaction of numerous heritable and environmental factors [16]. A plethora of genetic loci contribute to growth plate regulation and longitudinal bone growth [17]. While pathogenic variants affecting key growth-regulating pathways may lead to severe overgrowth syndromes and extreme tall stature, milder genetic variants may contribute to familial or constitutional tall stature, representing the upper end of the normal height distribution. The final growth phenotype is further modified by environmental (acquired) influences acting throughout childhood and adolescence [11].

According to the European Society of Pediatric Endocrinology (ESPE) classification, tall stature can be categorized as a primary growth disorder, a secondary growth disorder, or idiopathic tall stature [18]. This classification is consistent with the contemporary concept that disturbances affecting intrinsic or extrinsic regulators of growth plate function manifest as abnormal longitudinal growth [10]. Primary growth disorders are caused by genetic defects that directly affect growth plate function, whereas secondary growth disorders arise from factors extrinsic to the growth plate, including endocrine abnormalities such as GH excess, hyperthyroidism, and precocious puberty. In contrast, idiopathic tall stature encompasses familial and non-familial forms in which no identifiable pathological cause can be demonstrated despite detailed clinical evaluation [18]. Therefore, familial tall stature, idiopathic tall stature, syndromic overgrowth, and endocrine causes of excessive growth should be regarded as different phenotypic manifestations within the broader spectrum of stature abnormalities resulting from complex interactions among genetic, molecular, endocrine, and environmental determinants [11,18].

### 1.2. Clinical Assessment of Children with Tall Stature

The clinical evaluation of children presenting with tall stature aims to distinguish physiological variants of growth from pathological conditions associated with excessive linear growth. Tall stature is generally defined as a height exceeding two standard deviations above the mean for age and sex (>97th percentile), although children with a normal height who exhibit accelerated growth velocity or a predicted adult height substantially exceeding their genetic target height may also warrant further investigation [15,19,20]. The diagnostic approach begins with a thorough medical history, including prenatal and perinatal events, birth weight and length, developmental milestones, nutritional status, pubertal timing, medication use, and the presence of chronic diseases or endocrine disorders [15,20]. Particular emphasis should be placed on family history, including parental heights, pubertal development, and the presence of tall stature, overgrowth syndromes, or endocrine disorders among relatives, as familial tall stature represents the most common cause of non-pathological excessive growth [16,18,20]. Anthropometric assessment should include accurate measurements of height, weight, body mass index (BMI), head circumference, arm span, sitting height, and upper-to-lower body segment ratio, since disproportionate growth patterns may provide important clues regarding the underlying diagnosis [15,20]. Such measurements may facilitate the recognition of specific syndromic conditions associated with tall stature, including Marfan syndrome, Klinefelter syndrome, Sotos syndrome, Weaver syndrome and other genetic overgrowth disorders [9,21].

Physical examination should also focus on the identification of dysmorphic craniofacial features, skeletal abnormalities, cardiovascular manifestations, neurodevelopmental findings, and signs of endocrine dysfunction that may point to an underlying identifiable cause of excessive growth [15,20]. Growth should be assessed using standardized growth charts, with particular attention paid to growth velocity and percentile crossing, as accelerated growth may represent an early manifestation of endocrine disorders such as growth hormone excess, hyperthyroidism, or precocious puberty, as well as genetic overgrowth syndromes [15,16,20]. Bone age determination remains an essential component of the diagnostic work-up because it assists in the differentiation of constitutional variants from pathological causes of tall stature and contributes to the prediction of adult height [16,20]. Depending on the clinical findings, further investigations may include endocrine evaluation (serum T4, TSH, IGF-1, LH, FSH, testosterone, cortisol and prolactin levels, and glucose suppression test for GH), imaging studies, karyotype, and targeted molecular genetic analyses [11,15,18]. Recent advances in genomic technologies, particularly next-generation sequencing, have substantially improved the identification of monogenic causes of tall stature and overgrowth syndromes, enabling more accurate diagnosis [9,21]. As a consequence, current clinical genetics has shifted from a rigid deterministic framework to a nuanced probabilistic model. Navigating the complexity of overgrowth syndromes requires evaluating variant pathogenicity, variable clinical expressivity, and genotype–phenotype correlations. The framework of variant pathogenicity classification standardized by the of American College of Medical Genetics and Genomics is a useful interpretation approach for genetic findings. Therefore, the assessment of children with tall stature requires a multidisciplinary approach integrating clinical, radiological and genetic parameters, while careful evaluation of craniofacial morphology plays a crucial role in the diagnostic process, as it may provide clues regarding the underlying genetic etiology and guide subsequent cephalometric investigation [14,15,21].

## 2. Methods

The present scoping review was drafted using the Preferred Reporting Items for Systematic Reviews and Meta-analysis Protocols (PRISMAP). To identify internationally relevant documents, the electronic bibliographic databases MEDLINE and EMBASE were searched from 1980 to 15 May 2026. The search strategies were drafted by the senior experienced supervisor and further refined through team discussion. The search was conducted by the first author (N.Z.), who has relevant training and previous experience, under the supervision of C.Y. The final search results were exported into Microsoft Word, and duplicates were removed. The scientific articles that provided cephalometric data for genetic syndromes and conditions associated with tall stature or overgrowth were included (Supplementary Table S1). Excluded were reviews that either provided data irrelevant to the current topic or that were inaccessible online. The following keywords were utilized: tall stature cephalometry (8 results, which were all excluded as they provided no data regarding our study), overgrowth syndromes cephalometry (5 articles out of 14 were included) [22,23,24,25,26], gigantism cephalometry (6 articles out of 23 results were included) [23,24,27,28,29,30], hemihyperplasia cephalometric (no results), hemihypertrophy cephalometric (1 result out of 2 was included) [31], Simpson–Golabi–Behmel cephalometric (2 results, both of which were included) [23,32], Prader–Willi cephalometry (6 articles out of 12 were included) [33,34,35,36,37,38], Marfan cephalometry (15 results, 11 of which were included) [39,40,41,42,43,44,45,46,47,48,49] and Klinefelter cephalometry (6 articles were included out of 14 results) [50,51,52,53,54,55]. To increase consistency among reviewers, the same publications were screened, discussed the results and amended the screening and data extraction procedure before beginning the scoping review. Furthermore, there was evaluation of the titles, abstracts and text of all publications identified by initial search for potentially relevant publications. A data-charting form was developed to determine which variables to extract. Two reviewers independently charted the data, discussed the results with the supervisor and continuously updated the data-charting form in an iterative process. Data of article characteristics and contextual factors were abstracted, e.g., country of origin, type of knowledge user, type of engagement activity, use of a framework to inform the intervention. The studies were grouped by the types of disease they analyzed, and the type of settings, populations and study designs were summarized for each group. When a systematic review was identified, the number of studies included in the review was counted that potentially met the inclusion criteria and any studies possibly missed by our initial search were noted and evaluated.

## 3. Results

### 3.1. Molecular Pathways Associated with Tall Stature-Related Syndromes

Linear growth (defined as height) involves several cellular regulatory pathways related to regulation of gene transcription, genomic imprinting and intracellular vesicle trafficking. Mutations in genes involved in these pathways may lead to vastly different developmental syndromes, epigenetic imprinting disorders and cancer-related disorders. There are four main distinct molecular pathways whose dysregulation is associated with tall stature-related syndromes (Figure 1):

A. Nuclear factor I/X (NFIX) pathway

Nuclear factor I/X (NFIX) is a CCAAT-binding transcription factor that regulates networks of genes primarily involved in cellular differentiation, neural and skeletal development and oxidative stress response [56,57]. Loss-of-function mutations of the *NFIX* gene on chromosome 19p13.2 may lead to the disruption of NFIX-dependent transcriptional regulation, including RNA polymerase III transcription termination. pathways. Autosomal dominant mutations in *NFIX* are associated with Malan syndrome (OMIM 614753), which includes significant prenatal and postnatal overgrowth, macrocephaly, craniofacial deformities, intellectual disability, tall stature and advanced bone age [56,57,58,59].

B. Guanine Nucleotide-binding Protein Alpha Stimulating (GNAS complex locus) Pathway

Guanine Nucleotide-binding Protein Alpha Stimulating (*GNAS*) locus on chromosome 20q13.32 is a highly complex imprinted gene cluster that produces multiple RNA transcripts and signaling proteins [60]. It functions as the master regulator for hormone signaling and downstream effector pathways. Defects in *GNAS* imprinting can lead to hormone resistance (e.g., pseudohypoparathyroidism), while gain-of-function activating mutations lead to constitutive protein Gsα signaling and subsequent adenylate cyclase activity, cAMP/PKA activation and enhanced circulating GH levels and IGF-1-induced excessive linear growth and pituitary gigantism, particularly in McCune–Albright syndrome, which is also characterized by fibrous dysplasia and café-au-lait spots (OMIM 174800).

C. Small nuclear ribonucleoprotein polypeptide N (SNRPN) pathway

As a protein encoded by a gene located in an imprinted cluster on chromosome 15q11-q13 that is expressed almost exclusively from the paternal allele, small nuclear ribonucleoprotein polypeptide N (SNRPN) is a key protein involved in the spliceosome pathway and pre-mRNA splicing [61]. Loss of paternal expression or maternal uniparental disomy within the 15q11.2-q13, imprinted region, contributes to significant hypothalamic and metabolic impairments, characteristic of Prader–Willi syndrome (OMIM 176270). Patients with that syndrome are characterized by impaired GHRH/GH signaling, initially a significantly short stature and reduced lean mass, subsequently increased food intake and obesity, thereby leading to an overgrowth-related phenotype.

D. Vacuolar protein sorting-associated 13B (VPS13B) pathway

Vacuolar protein sorting-associated 13B (VPS13B), also known as Cohen syndrome protein 1 (COH1), is a large peripheral Golgi membrane protein encoded by *VPS13B* gene on chromosome 8q22.2 [62]. It plays a central role in lipid transport and vesicle-mediated intracellular protein sorting. Loss-of-function mutations in the *VPS13B* gene lead to the impairment of Golgi organization, vesicle trafficking, protein sorting, as well as glycosylation. All the above are associated with developmental abnormalities of autosomal recessive Cohen syndrome (OMIM 216550), including intellectual disability, microcephaly, distinct facial features, initial growth impairment and subsequent rapidly occurring obesity, leading to an overgrowth-related phenotype.

### 3.2. Cephalometric Assessment of Children with Tall Stature

Lateral cephalometric radiography is a standardized, widely available, low-dose imaging method routinely used by dentists to estimate craniofacial dimensions [63,64,65]. This radiographic technique is used extensively in oral and maxillofacial surgery and orthodontics in order to assess anomalies in the maxillofacial region. The lateral cephalometric radiograph is the most commonly utilized method (Figure 2) and is able to diagnose anomalies of the craniofacial complex in both the sagittal and vertical planes [66]. Angular and linear measurements obtained through conventional cephalometric analysis, or direct use of landmark coordinates in more recently developed geometric morphometric approaches, provide information about the quantitative extend of the maxillofacial discrepancies and could be a useful tool in assessing the craniofacial phenotype in children with tall stature. Cephalometric analysis in children with distinct craniofacial proportions who do not yet display clear somatic overgrowth may provide the basis for early diagnosis and potential therapy. In addition, changes in the bony architecture and jaw–base relationships that occur over time may be crucial for predicting mandibular overgrowth and planning early orthodontic, orthognathic or other treatment interventions.

Cephalometric analysis may be very useful in objective phenotyping of subtle dysmorphic features by providing precise measurable linear and angular values that can be compared to age- and sex-matched normative data. In children with short stature and genetic syndromes, cephalometric analyses have identified patterns such as posteriorly positioned jaws and vertical facial growth, highlighting the potential of this tool to contribute to phenotypic definition and to support clinical suspicion for specific genetic diagnoses [13]. Similarly, systematic cephalometric characterization of children with tall stature of genetic etiology (particularly those with GH excess-related pituitary disease) may uncover reproducible craniofacial patterns that facilitate differentiation between constitutional tall stature, monogenic overgrowth syndromes, and pituitary causes, thereby contributing significantly to the multidisciplinary management in pediatric endocrinology, orthodontics, and clinical genetics.

### 3.3. Cephalometric Data for Syndromes Associated with Tall Stature

A plethora of genes is implicated in the determination of linear growth velocity and final adult stature. Genetic defects affecting those genes can manifest as abnormally tall stature. The pathogenetic mechanism through which such mutations lead to high stature include direct EGP activation, induction of connective tissue hyperplasia and excessive GH production [67]. The phenotypes of high stature disorders are determined not only by the underlying molecular defect but also by the developmental timing of its pathogenic activation. The temporal window during which the pathological process takes place may profoundly influence the resulting growth pattern.

In several overgrowth syndromes, accelerated growth is confined to fetal life or early childhood and is frequently connected with advanced skeletal maturation, followed by subsequent normalization or deceleration of linear growth and, in some cases, a normal or even reduced final adult height [68]. This is characteristic of disorders such as Beckwith–Wiedemann syndrome, Prader–Willi syndrome, Cohen syndrome and Marshall–Smith syndrome. Moreover, disorders characterized by growth hormone (GH) excess including McCune–Albright syndrome, multiple endocrine neoplasia type 1 (MEN1), and, less commonly, multiple endocrine neoplasia type 4 (MEN4), illustrate how an identical endocrine disturbance may give rise to distinct clinical phenotypes depending on the temporal window of pathogenic activation [60,69]. GH hypersecretion occurring before epiphyseal growth plate fusion results in excessive longitudinal bone growth and manifests clinically as gigantism, whereas the same pathological process developing after epiphyseal fusion primarily promotes periosteal bone apposition, skeletal thickening and soft tissue overgrowth, producing the phenotype of acromegaly. Thus, gigantism and acromegaly represent age-dependent manifestations of the same endocrine disorder proving the principle that the developmental timing of a pathogenic event is a critical determinant of the ultimate overgrowth phenotype [70].

#### 3.3.1. Gh Excess (Acromegaly/Gigantism)

In a study including 14 subjects with acromegaly, common features included increased growth of the soft tissue of the tongue, the soft palate and the pharynx. More specifically, there was a positive correlation between growth hormone and soft tissue growth. The subjects exhibited increased tongue length, increased length and height of soft palate, increased mandibular plane-hyoid bone distance and increased thickness of vocal tract [26].

In another study, cephalometric measurements were obtained from rodents after the induction of pituitary gigantism and acromegaly by administration of growth hormone at different ages. The skull length increased greatly in younger rats and plateaued in older ones. The skull length gains were almost entirely in the facial cranium, with an increased bizygomatic width in both injected groups. The neurocranium gained no length and widened only slightly. Palatal and dental growth followed facial patterns in both groups. Cranial vault bones thickened and, in older rats, developed surface irregularities, giving them a more massive, acromegaloid structure [30]. These findings are consistent with two patients with GH excess, in which the head circumference was normal, but the exhibited increased facial widths, greatly increased facial heights, longer clivus and a normal cranial base angle [29].

#### 3.3.2. Marfan Syndrome

Marfan syndrome is inherited in an autosomal dominant mode, and its features include disproportionate tall stature and aortic dilation. It is caused by heterozygous mutations in the fibrillin 1 gene (*FBN1*). Fibrillin 1 is an extracellular matrix glycoprotein implicated in bone metabolism via interactions with the transforming growth factor beta (TGF-β) signaling pathway [10]. The mutated protein is unable to sequestrate the signaling pathway, resulting in a prolonged signaling during the growth period and contributes to the characteristic phenotype of the syndrome.

Cephalometric analysis is an important diagnostic tool in the early detection of the syndrome, as affected individuals exhibit a high prevalence of craniofacial abnormalities [44]. Common findings include constriction of dental arches, high and narrow hard palate [47], posteriorly placed maxilla and mandi [42]. Among 76 affected individuals referred for orthodontic treatment, the most common findings included crowded teeth, maxillary overjet and open bite. Patients also exhibited a high and narrow palatal vault as well as posteriorly placed maxilla and mandible [39].

In a study including 15 patients, significant features included posteriorly placed maxilla and mandible, reduced maxillary length, increased total anterior facial height with an increased lower anterior facial height, obtuse gonial angle, steep mandibular plane, reduced posterior nasal airway height and space and an increased distance from the mandibular plane to the hyoid bone. Also, the severity of craniofacial abnormalities was concomitant with the severity of obstructive sleep apnea in these patients [46]. Reduced horizontal airway dimensions and increased vertical airway length were also reported in 30 individuals with Marfan syndrome [41].

In another study with 45 affected individuals, common features were posteriorly placed maxilla and mandible, increased facial height and highly arched palate [49]. Other phenotypic traits of the syndrome include dolichofacial shape, nasal and alar deviation, abnormally accentuated mentolabial fold, incompetent lips and gingival smile [40].

In a study of 26 Marfan syndrome individuals, both the anterior and posterior cranial base lengths were reduced. This reduction could be accounted for deficient sutural growth. Affected individuals also presented an increased anterior facial height, as a result of a reduction in anterior upper facial height and an increased anterior lower facial height. The latter may, amongst others, be closely associated with vertical midface hypoplasia. Posteriorly placed maxilla and mandible, reduced maxillary length, and a posterior rotation of the mandibular plane were evident in most of the patients. Palatal height and length were significantly increased [45].

Lastly, a case study described a 28-year-old female patient. She exhibited posteriorly placed maxilla and mandibular deficiency and was undergoing orthodontic retreatment, after maxillary first premolar extractions with a maxillary surgical procedure and a genioplasty lead to a relapse [48].

#### 3.3.3. Loeys–Dietz Syndrome

Loeys–Dietz syndrome (LDS) is a rare autosomal dominant connective tissue disorder with multisystemic involvement, resulting from heterozygous mutations in the TGF-β signaling pathway [71,72]. LDS and Marfan syndrome share common phenotypic features; however, patients with LDS have more severe vascular involvement. Mutations along the TGF-β pathway are implicated in the pathogenesis of LDS, including the genes encoding TGF-β receptor 1 (*TGFBR1*) and TGF-β receptor 2 (*TGFBR2*), mothers against decapentaplegic homolog 3 (*SMAD3*), TGF-β ligand 2 (*TGFB2*) and TGF-β ligand 3 (*TGFB3*), considered LDS types 1–5 respectively [73].

LDS is characterized by the following triad of features: craniofacial manifestations, vascular anomalies and craniofacial manifestations. The craniofacial features include craniosynostosis, facial asymmetry, blepharoptosis, strabismus, hypertelorism, posteriorly positioned maxilla and mandible, flat midface, malar hypoplasia, dolichocephaly, blue sclerae, temporomandibular joint disorders and obstructive sleep apnea. The degree of craniofacial involvement varies between the different types of LDS. The craniofacial alterations from most severe to least severe were identified in LDS2, LDS1, LDS5, LDS3 and LDS4 subtypes. Cephalometric analysis demonstrated an increased cranial base angle with reduced anterior cranial base length and underdevelopment of the maxilla and mandible, with reduced pharyngeal airway space. In the vertical plane, there was reduced anterior facial height and posterior facial height. The lower facial height, ramus height and gonial angle were within normal range. In the sagittal plane, the midface length was significantly reduced. Moreover, the mandibular body length was reduced in all LDS subtypes, except for LDS5, where it was significantly increased. The aforementioned manifestations could be attributed to a more inferior location of the sella turcica, which contributed to an alteration in the inclination of the anterior cranial base, as well as to the underdevelopment of both the maxilla and the mandible [72,73].

#### 3.3.4. Sotos Syndrome

Sotos syndrome is an overgrowth disorder with prenatal onset and is inherited in an autosomal dominant manner. The condition occurs through mutations in the *NSD1* gene or through deletion in the 5q35 region that includes the *NSD1* gene. NSD1 is a transcriptional coregulator protein that contains multiple important domains that enable the protein to methylate histones (SET domain) or bind both methylated (PHD domains) and demethylated histones (PWWP domains) [68]. The protein also interacts with nuclear hormone receptors. Missense variants in patients with Sotos syndrome have been found in the SET, PWWP, and PHD domains of NSD1, suggesting all three functional domains are important for the regulation of body growth [10,68]. Sotos syndrome is also associated with decreased activity of the Ras/MAPK pathway, which regulates proliferation and differentiation of growth plate chondrocytes [10].

In a published study, two adolescent patients demonstrated metopic protrusion, high and narrow hard palate, posteriorly positioned maxilla and mandible, the one presenting skeletal class III relationship and the other skeletal class II, leptoprosopic face and v-shaped mandible. Upon reexamination both patients presented an increase in lower facial height and decreases in facial axis and depth. Increased facial height without mandibular forward overgrowth is a craniofacial feature of the syndrome [24]. In another study including 14 children, the maxillofacial region was characterized by an increased facial height, increased occipitofrontal head circumference and relative prognathism [74]. Three other patients exhibited oxycephaly, a steep anterior cranial base angle and anteriorly positioned maxilla and mandible [27].

#### 3.3.5. Simpson–Golabi–Behmel Syndrome

Simpson–Golabi–Behmel syndrome is characterized by tall stature, is inherited in an X-linked manner and is caused by mutations in the *GPC3* (type 1) or *OFD1* and *PIGA* genes (type 2). Two children demonstrated increased anterior cranial base length and increased length of the jaws [13,23,32]. The former subject exhibited a class I skeletal pattern and hyperdivergent skeletal planes with increased lower anterior facial height and increased gonial angle [32], while the latter had a skeletal class III pattern and an increased lower anterior facial height [23].

#### 3.3.6. Klinefelter Syndrome

Klinefelter syndrome is a sex chromosome aneuploidy (XXY usually) characterized by tall stature. The syndrome’s craniofacial morphology is a result of selectively inhibited cranial growth. It is supported that an extra X chromosome in men leads to increased cranial base flexion, anteriorly placed maxilla, profound mandibular prognathism and abnormalities in the sagittal plane, while skeletal class III jaw relationship is a common finding [50,55].

In a study, 35 patients exhibited reduced angles between the occlusal plane and the cranial base and between the occlusal plane and the Frankfort horizontal plane. These reduced cephalometric parameters are attributed to the impaired growth of the cranial base and the greater growth of the lower border of the mandible, driven by the extra X chromosome [51].

In another study involving 28 males with Klinefelter syndrome, the cardinal maxillofacial features included reduced anterior cranial base length, reduced cranial base angle, reduced anterior and posterior facial height, reduced maxillary length, anteriorly placed maxilla which stood almost parallel to the anterior cranial base, reduced ramus length, anteriorly positioned mandible and skeletal class III jaw relationship [54].

Additionally, 35 individuals with Klinefelter syndrome presented reduced cranial base length and cranial base angle, protruding nasal bones, increased length and anterior placement of both the maxilla and the mandible and increased gonial angle [52]. In accordance with the aforementioned findings, in another 22 individuals with Klinefelter syndrome, the anterior cranial base, anterior and posterior facial height were reduced, but the length of the maxilla and the ramus were also significantly decreased [53].

#### 3.3.7. Hemihypertrophy

Hemihypertrophy is a condition characterized by unilateral overgrowth of the body with or without unilateral hypertrophy of the face, in which both the hard and soft tissues are involved. Asymmetric overgrowth of the body is a feature of Beckwith–Wiedemann syndrome [75], but in some cases the cause remains unknown. Dental findings include premature eruption and development of the teeth, macrodontia, malocclusion and congenitally missing teeth.

An individual with facial hemihypertrophy and no family history of this condition demonstrated unilateral tongue enlargement with hypertrophic fungiform papillae. In addition, the soft and hard tissues of the affected side, such as the lips, palate, maxilla and mandible, were also unilateraly overgrown resulting in facial asymmetry and severe malocclusion of the permanent dentition [31].

#### 3.3.8. Beckwith–Wiedemann Syndrome

Beckwith–Wiedemann syndrome (BWS) is a condition associated with pathologic overgrowth and multiple congenital malformations, including macroglossia, visceromegaly and embryonal tumors [75]. The disorder is characterized by overgrowth in the early stages of life, but stature seems to normalize in adult life and final tall stature is present in 44% of adult patients [76]. The pathogenesis of the syndrome involves epigenomic and genomic changes in the imprinting clusters on chromosome 11p15. This genetic locus contains genes such as *IGF2*, *H19*, *KCNQ1OT1* and *CDKN1C*, the expression of which depends on the maternal or paternal origin of the gene [75]. Studies support that conceptions achieved through assisted reproduction exhibit excessive differences in DNA methylation, and this could also be implicated in the pathogenesis of the syndrome [68].

One of the most prominent maxillofacial features of the syndrome is excessive macroglossia. Constant tension applied from the tongue has a thrusting effect on dentoalveolar structures causing protrusion, particularly in the mandible. This results in an anterior cross-bite. Additionally, the gonial angle is increased leading to an increase in the effective length of the mandible. Nevertheless, a study reported that changes in the maxillofacial complex undergoing later during the growth period, such as elongation of the base of the tongue and posterior and inferior placement of the hyoid bone, seem to allow for the accommodation of the tongue within the oral cavity [77]. In contrast to that, another study supported the notion that macroglossia is not implicated in the post-natal mandibular overgrowth in BWS and that mandibular overgrowth resulting in class III skeletal relationship is part of the syndrome [25]. Both studies, however, concluded that early partial glossectomy that was applied in the past does not change the maxillofacial outcome and should be delayed or even abandoned [25,77].

Another study indicated that BWS patients exhibit normal mandibular length and increased mandibular width and altered Wits values of cephalometric measurement [78]. In another recent study including 25 children, 76% exhibited hyperdivergent skeletal planes with dental class I relation. About 40% had an anterior open bite and 16% also showed unilateral posterior cross-bite.

#### 3.3.9. Prader–Willi Syndrome

Prader–Willi syndrome pathogenesis includes loss of specific paternally expressed genes on chromosome 15q11.2-q13. This chromosomic region includes a plethora of genes, such as *SNORD116*, *AGEL2*, *NECDI*, *NIPA1*, *NIPA2*, *CYF1P1*, *GCP5*, *MKRN3*, *MAGEL2*, *NECDIN*, *SNURF-SNRPN*, C/D box snoRNAs, *UBE3A*, *ATP10A*, *GABRB3*, *GABRA5*, *GABRG3*, *OCA2* and *HERC2*. This loss occurs through paternal deletion (65–75%), maternal uniparental disomy (20–30%) and imprinting defects (1–3%). Children with PWS demonstrate hyperphagia and obesity, but they exhibit short stature, due to growth hormone deficiency [61].

In a cohort of 42 Prader–Willi syndrome (PWS) patients, 28% exhibited skeletal class II jaw relationship due to a more posteriorly placed mandible and downward facial growth with increased anterior height, 24% presented with a decreased cranial base angle added to the aforementioned findings, whereas 48% subjects exhibited skeletal class III jaw relationship due to an anteriorly placed mandible with horizontal facial growth and abnormal maxillary and mandibular incisors inclination [13,33]. In another study, 20 patients with PWS presented with decreased linear parameters in their lateral cephalometric radiographies (maxillary and mandibular length, as well as posterior and anterior facial heights) and almost normal linear measurements. The frontal cephalometric X-rays of these patients exhibited decreased maxillary and mandibular horizontal dimensions and a reduced interzygomatic distance [13,36]. The aforementioned findings are in accordance with the measured cephalometric parameters in another group of PWS subjects. The measured skeletal parameters, such as maxillary and mandibular length, ramal height and posterior facial height were decreased, while the covering soft tissues were enlarged [13,34].

In a case of a 10-year-old girl with typical PWS, a confined growth of both the maxilla and the mandible was also observed [35]. Other craniofacial manifestations included an increased bony interorbital distance and a lower position of the cribriform plate of the ethmoid bone [38].

In a cohort of 18 PWS patients there is a reduction in the majority of the measured craniofacial parameters, but the degree of this reduction demonstrates high variation from subtle anomalies to severe disturbances among affected individuals. In the horizontal plane, PWS subjects present reduced values of the angular parameters and increased values of the linear measurements. In the vertical plane all the parameters were shorter. The linear measurements of the mandible and maxilla, with the exception of the palatal length, showed shorter dimensions in the PWS group. The mandibular plane angle and the gonial angle were reduced in PWS patients. The angles evaluating the interrelationship between maxilla and cranial base, mandible and cranial base and sagittal interrelationship between the maxillary and mandibular apical bases were wider in PWS patients [37].

#### 3.3.10. Cohen Syndrome

Cohen syndrome is inherited in an autosomal recessive manner and is caused by mutations in the VPS13B gene. The syndrome is characterized by truncal obesity and short stature [62].

In a study with 14 patients with Cohen syndrome common craniofacial manifestations include reduced anterior and posterior cranial base lengths and normal cranial base angles. The dimensions of foramen magnum, posterior facial heights and the anteroposterior position of the jaws were within normal limits. The patients also presented reduced head size (microcephaly), labially inclined upper incisors and reduced upper lip length [79].

## 4. Discussion

In contrast to growth failure, children with tall stature account for a smaller proportion of referrals to pediatric endocrinology clinics. Despite the fact that constitutional tall stature accounts for the majority of cases, meticulous evaluation of each individual is crucial, because of the possible existence of an underlying endocrine, neoplastic, or genetic disease [7]. Adding to its complexity, the phenotypic manifestations of high stature disorders are determined not only by the underlying molecular defect, but also by the temporal window during which the pathological process acts.

This scoping review presents the currently available data on lateral cephalometric characteristics of children with tall stature of genetic etiology and discusses briefly the craniofacial complex deviations from norm and the underlying molecular mechanisms that are affected (Supplementary Table S2). Despite the limited number of existing reports, their consensus agreement supports the notion that lateral cephalometric radiography could potentially serve as a promising tool for early recognition and improved genetic evaluation of pediatric tall stature as well as for phenotype–genotype comparative research of conditions associated with tall stature.

Currently, the vast majority of papers applying cephalometric analyses use conventional angular and linear measurements, as commonly applied in clinical orthodontics. Such measurements present with several limitations: they do not provide a comprehensive description of shape, leading to conflicting conclusions, are confounded by bias regarding the reference structures (e.g., the anterior cranial base or the Frankfurt horizontal), and are highly inter-correlated, making it difficult to obtain reliable statistical results. Progress in geometric morphometric methods (GMMs) has mitigated these problems, but adoption of GMMs, such as Procrustes alignment and Principal Component Analysis, has been slow [63,66,73,80,81,82].

In several syndromes, such as Beckwith–Wiedemann syndrome, Prader–Willi syndrome, Cohen syndrome and Marshall–Smith syndrome, overgrowth occurs early in life and is connected with advanced skeletal maturation, followed by subsequent normalization or deceleration of linear growth and, in some cases, a normal or even reduced final adult height [68]. Moreover, disorders characterized by growth hormone excess, such as McCune–Albright syndrome, multiple endocrine neoplasia type 1, multiple endocrine neoplasia type 4, indicate how an identical endocrine disturbance may give rise to distinct clinical phenotypes, depending on the temporal window of pathogenic activation, manifesting clinically as gigantism or acromegaly [60,69,70].

Several characteristics of geometric morphometric methods are particularly suited for describing and evaluating the phenotype of the craniofacial skeleton. Although there are several anatomic landmarks that can be accurately located on the cephalogram, some lying on intersections of bony outlines and sutures (e.g., nasion), others on local extremes of curvature (e.g., anterior nasal spine) and others at the center or edge of circular structures (e.g., sella and porion), large extents of surface outlines, such as the mandibular border, do not have any discernible landmarks. Such outlines can be studied morphometrically by drawing curves along them and then placing sliding semilandmarks, which adapt their position along the curves, based on the overall shape, thus achieving better anatomic correspondence between specimens [83]. Combined with Procrustes alignment, which removes the confounding effect of size, sliding semilandmarks can reveal subtle patterns of variation and shape differences not otherwise evident by conventional cephalometrics. Alternatively, size can be integrated with shape data to study allometric effects, particularly relevant to cases with overgrowth or size-related conditions. GMM have been applied successfully to show differences in craniofacial shape in patients with tooth agenesis, cleft lip and palate, and beta thalassaemia, among others [66,80,81,82,84]. Geometric morphometrics also supports three-dimensional diagnostic data, if available (e.g., computed tomography) [85], allowing analysis of phenotypic variation that is not accessible by planar 2D radiography.

As such, cephalometry seems to be potentially ideal for the early diagnosis of the above-mentioned genetic and endocrinological conditions, as the craniofacial region is susceptible to both hormonal and genetic influences [12]. This is evident in cases of GH excess, where facial soft tissue hyperplasia, enlarged facial dimensions, mandibular prognathism and dental diastemata are common features. Accordingly, studies in children with short stature of genetic etiology show that growth failure is paralleled by changes in craniofacial morphology [13]. Consequently, if quantitative data in large series of subjects become available and craniofacial patterns arise for certain syndromes or categories of stature anomalies, e.g., genetic or endocrine, then utilization of the lateral cephalometric radiography in conditions associated with excessive linear growth could serve as a useful tool for the early detection of a broad range of growth abnormalities.

The rarity of pediatric GH excess and the scarcity of existing craniofacial data in overgrowth conditions create the need to synthesize the current evidence in order for a relationship to be established between certain pathological conditions and specific craniofacial phenotypes. Prolonged exposure to excessive levels of GH and IGF-1 may lead to serious systemic, metabolic and cardiovascular complications and therefore early diagnosis is important [14]. Thus, the lateral cephalometric radiography could be implemented in the early detection of such conditions, before the adverse effects become irreversible.

Nevertheless, cephalometric analysis is obviously not a unique and self-contained diagnostic tool for genetic diseases. It is well known that many syndromes display overlapping phenotypes, making exact morphological diagnosis challenging based on radiographs alone. Instead, cephalometric data may be best utilized as a phenotypic screening step that prompts further clinical evaluations, such as assessing bone age, conducting genetic investigation including a karyotype or a targeted multigene panel testing. The most beneficial management of craniofacial syndromes requires a multidisciplinary approach where experts from pediatrics, genetics, dentistry, orthodontics, oral maxillofacial surgery, neurosurgery, psychology and speech therapy should collaborate to create a unified treatment plan specifically designed for each particular case.

Our scoping review has some limitations. To make our review feasible, we were only able to include a sample of publications from the MEDLINE and EMBASE databases that were publicly available. Further adding to this issue is that our results are only up to date as of July 2026, the time of submission of our review. As such, the enrichment of the published data on lateral cephalometric characteristics in children with tall stature of genetic etiology is required in order to assess the potential use of these craniofacial traits as markers for earlier recognition and improved genetic evaluation of pediatric cases with extreme stature phenotypes [13,14].

Systematic cephalometric evaluation of children with tall stature of genetic etiology may format reproducible craniofacial patterns that facilitate differentiation between constitutional tall stature, certain monogenic overgrowth syndromes, and pituitary causes, thereby contributing significantly to the multidisciplinary management in clinical genetics, endocrinology, and orthodontics. The current lack of evidence to support cephalometric evaluation of children with tall stature appears to pose a challenge. The aim of this scoping review was to identify gaps in the literature which may guide a future systematic review. However, the lack of evidence found means that undertaking a systematic review is not appropriate. This advocates high-quality research being needed to determine whether cephalometry may be of benefit for this population, especially in difficult to diagnose cases, for example, when a genetic variant detected is a Variant of Unknown Significance (VUS). It may be envisaged that in the future large cohort studies with genotype–phenotype correlations could propose cut-off values for cephalometric characteristics of certain conditions with tall stature, enabling clinicians to better diagnose and treat some genetic disorders based on their patients’ craniofacial phenotype.

## Figures and Tables

**Figure 1:**
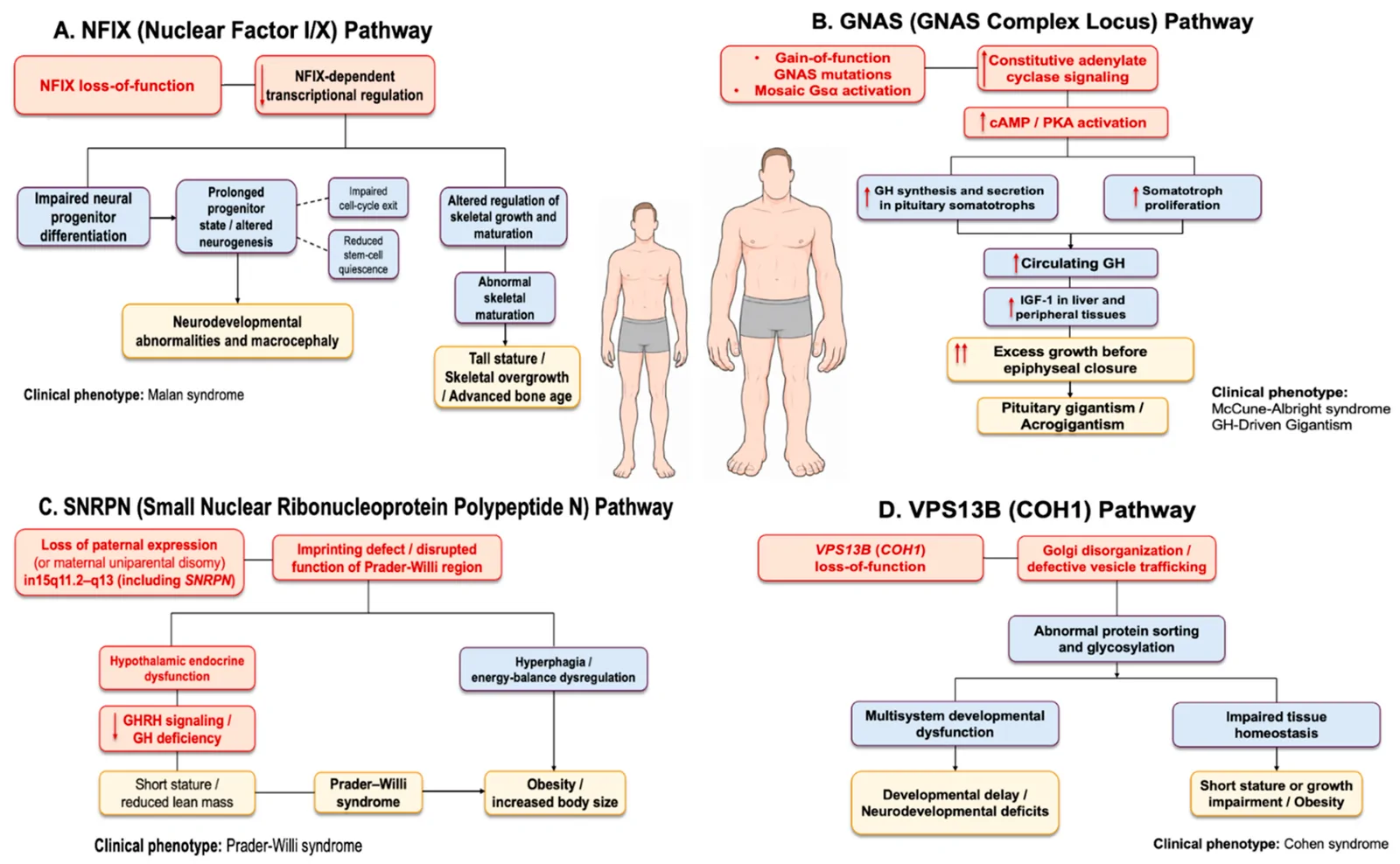
Diagrammatic overview of the key molecular pathways whose dysregulation is associated with abnormal growth phenotypes and tall stature-related syndromes. The figure summarizes four distinct mechanisms through which the *NFIX*, *GNAS*, *SNRPN* and *VPS13B* genes influence growth regulation and whose dysregulation results in overgrowth-related clinical phenotypes described in text. Upward arrows indicate an increase in gene or protein expression, molecular activity, signaling, concentration or secretion, whereas downward arrows indicate the reduction or inhibition of the corresponding parameters.

**Figure 2:**
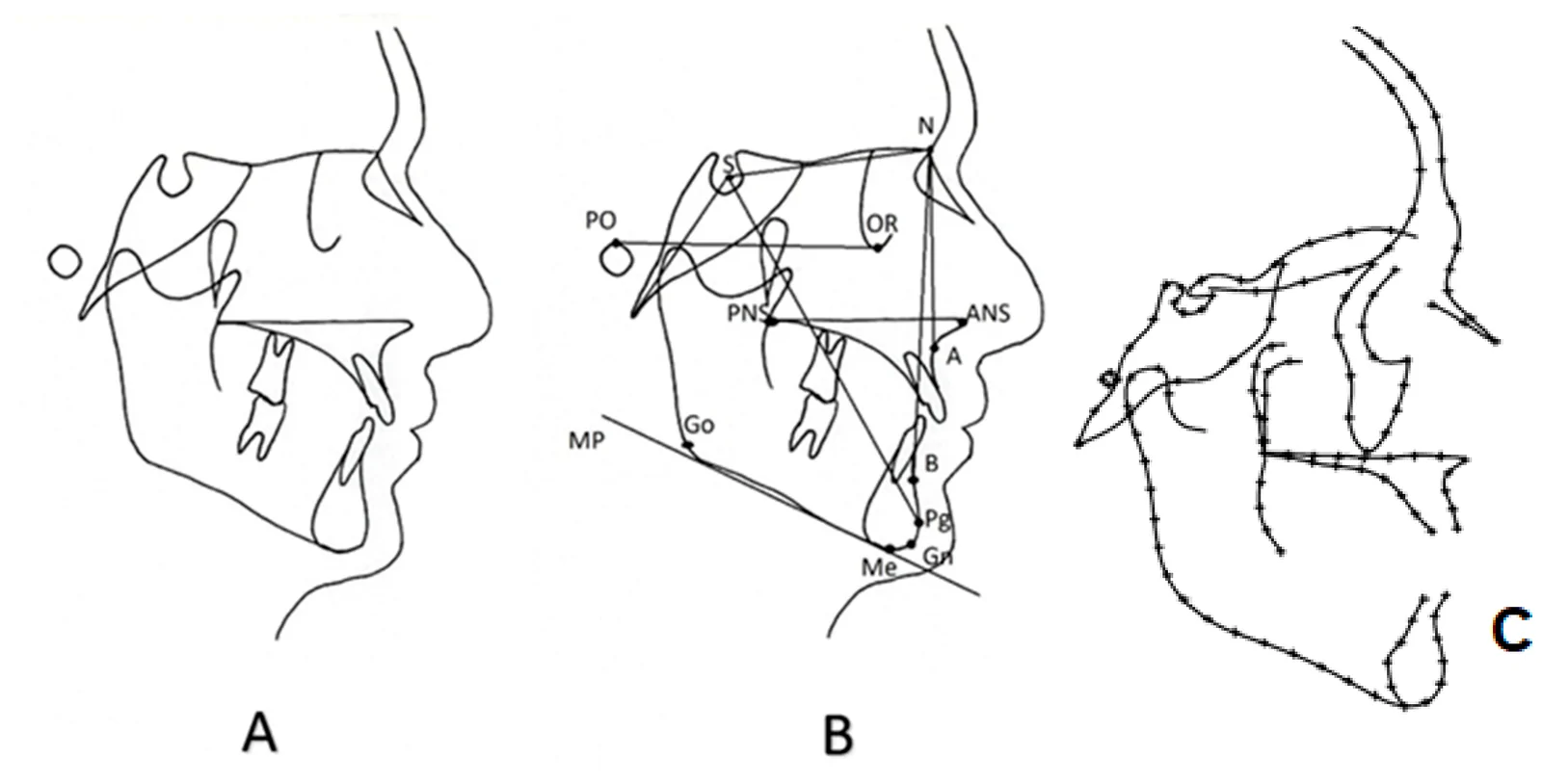
Schematic representation of lateral cephalometric analysis. (**A**) Cephalometric tracing. (**B**) Most utilized cephalometric landmarks and reference planes corresponding to lines connecting the landmark points. A: A-point; ANS: anterior nasal spine; B: B-point; Gn: gnathion; Go: gonion; GoMe: mandibular plane; Me: menton; N: nasion; NA plane; NB plane; NPg: facial plane; OR: orbitale; PNS: posterior nasal spine; Pg: pogonion; PNS-ANS: palatal plane; Po: porion; PoOr: Frankfurt horizontal plane; S: sella; SN: anterior cranial base plane; *Y*-axis SPg or SGn. (**C**) Geometric morphometric approach, using a large number of sliding semilandmarks to describe shape of osseous outlines.

## Data Availability

The original contributions presented in this study are included in the article. Please direct any additional inquiries to the corresponding author.

## Supplementary Materials

The following supporting information can be downloaded at https://www.mdpi.com/article/10.3390/genes17101167/s1. Supplementary Table S1. Search strategy. Supplementary Table S2. Preferred Reporting Items for Systematic reviews and Meta-Analyses extension for Scoping Reviews (PRISMA-ScR) Checklist. Reference [86] are cited in the Supplementary Materials.
